# “We have that strong R, you know”: the enregisterment of a distinctive use of rhotics in Santomean Portuguese

**DOI:** 10.1515/ijsl-2021-0099

**Published:** 2023-01-27

**Authors:** Marie-Eve Bouchard

**Affiliations:** Department of French, Hispanic and Italian Studies, The University of British Columbia, Vancouver, BC, Canada

**Keywords:** diaspora, enregisterment, indexicality, language contact, rhotics, Santomean Portuguese

## Abstract

This study examines how the use of rhotics in Santomean Portuguese is becoming enregistered as a feature that marks Santomeans’ national identity. It is based on ethnographic fieldwork and semistructured interviews with Santomeans living on São Tomé Island and in Portugal. The qualitative analysis of the data reveals the process that leads to the use and awareness of the rhotic feature among Santomeans. This increasing awareness is analysed in terms of orders of indexicality. The author suggests that awareness of this rhotic feature among Santomeans is contingent on having contact with Portuguese speakers of non-Santomean origin, as they only become aware of their distinctive use of rhotics when they are in contact with speakers of another variety of Portuguese on the island, in the diaspora, or online. Also, even if this feature is perceived negatively by many, it remains available for identity-driven use to express a connection to São Tomé and Príncipe.

## 1 Introduction

*Estão sempre a dizer que a gente parece com franceses que estão sempre a carregar no R.*

They always say that we sound like French people who pronounce their R’s strongly.

(Clara, a young Santomean woman living in Portugal)

I met Clara in 2015 when I was conducting ethnographic fieldwork on São Tomé Island for the first time. I interviewed her as part of my study on the languages of São Tomé and Príncipe. I did not yet know that the use of rhotics would become central to my research, but Clara was a typical strong-R user, with a frequent use of uvular fricatives in her speech. Two years later, Clara moved to a small town in Central Portugal (*Região do Centro*) to attend a professional high school and study marketing. In 2019, when I decided to do field research among the Santomean diaspora in Portugal, I contacted her. Clara was then 21 years old and had been living in Portugal for two years. When I first interviewed Clara in São Tomé City, I asked her about the distinctive features of Santomean Portuguese that differentiate it from European Portuguese. Her answer included intonation, lexical items, and grammatical errors – the pronunciation of r-sounds was not brought up. But four years later, when asked the same question, Clara answered that the pronunciation of r-sounds and a fast speaking rate were the two main distinctive features of Santomean Portuguese. She had become aware of her distinctive use of r-sounds, mainly because the Portuguese in her surroundings had told her repeatedly that she used them differently (as she mentioned in the interview excerpt above). This is not surprising, since for non-Santomeans, one of the most distinctive features of the variety of Portuguese spoken on São Tomé Island is the pronunciation of /r/ as a voiced uvular fricative [ʁ] (or sometimes a trill [r]) in contexts where, phonologically, a flap [ɾ] is expected in most other varieties of Portuguese spoken in Brazil and Portugal (Bouchard 2019a). Even though the rhotics show great variability in the Portuguese-speaking world (cf. Veloso 2015; Rennicke 2015), this pronunciation of /r/ is quite unique among the varieties of Portuguese.1The dialect of the fishermen of Setúbal, a city situated 48 km south of Lisbon, is characterized by its use of the voiced uvular fricative [ʁ] for all instances of /r/ (Veloso 2015: 333). More sociolinguistic studies on this dialect are necessary.


**Figure 1:**
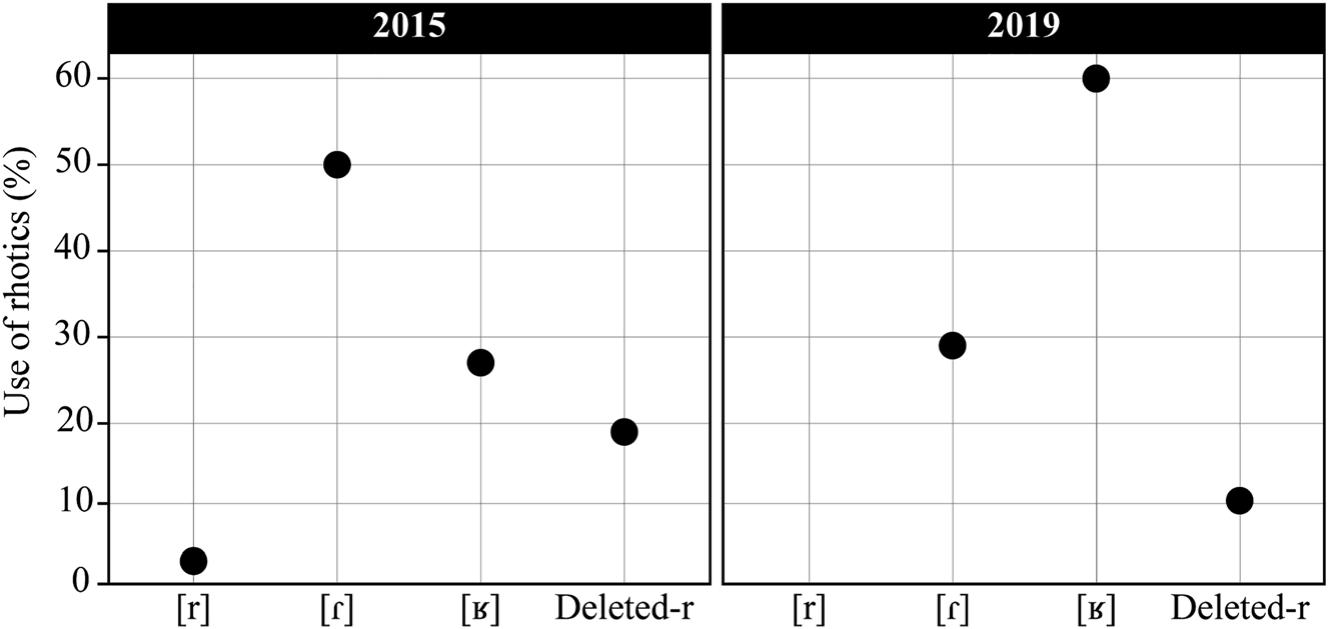
Clara’s use of rhotics (in all phonological contexts) in 2015 and in 2019.

One of the first things I noticed when I reencountered Clara in 2019 was that she had not converged toward a more standardized use of rhotics during her few years stay in Portugal, even though she had been in everyday contact with European Portuguese and was being schooled in this variety. Her use of rhotics actually showed the opposite trend; she had become even more of a strong-R user. The results in Figure 1 confirm my observations: Clara was using more uvular fricatives and less flaps in her speech in 2019 compared to 2015. This data comes from two interviews conducted with Clara and consist of tokens of rhotics (*N *=* *120 in 2015 and *N *=* *110 in 2019) that were extracted from the middle of the interviews, from all phonological contexts of the words (i. e., syllable-initial, intervocalically, second position of onset, and coda position). The tokens of rhotics were analysed using Praat (Boersma and Weenink 2017).

Clara’s case brought up many questions about language use, identity, and how certain linguistic features that were once entirely unnoticed by the speaker become noticeable and associated with social values. For this reason, this study examines ethnographic and interview data to determine how the use of rhotics in Santomean Portuguese has become (and is still becoming) enregistered, and has come to index a certain identity. I address the following questions: how do the ways of pronouncing rhotics move from being an unnoticed feature of the young urban Santomeans to being a noticeable feature associated with Santomean identity? How is the Santomean variety of Portuguese enregistered in terms of metalinguistic commentary? To answer these questions and to see how linguistic features and social meaning can be linked, I use the concepts of indexicality and enregisterment (cf. Silverstein 2003; Agha 2003, 2007; Johnstone et al. 2006). With this framework, I aim to demonstrate that the distinctive use of rhotics has indexical links to Santomean national identity and that it is enregistered in Portugal as a Santomean feature.

In the next section of this article, I set the requisite background for this study by examining previous works on the use of rhotics among Santomeans on São Tomé Island and in Portugal. In Section 3, I offer a brief presentation of the main theoretical concepts that inform this study and a description of the methodology used to collect data. In Section 4, I analyse the processes of enregisterment of r-sounds and how have they led to the emergence of dialect awareness among Santomeans. Finally, the conclusion synthesises the findings and points out the importance of contact with non-Santomeans for the enregisterment of the rhotic feature.

## 2 Background

To begin, I provide the relevant background on two fronts: the use of rhotics on São Tomé Island, and the use of rhotics among the diaspora in Portugal.

### 2.1 Rhotics in Santomean Portuguese

The use of rhotics is a salient feature in the variety of Portuguese spoken in São Tomé and Príncipe and it challenges the distribution rules that have been taken for granted elsewhere in the Portuguese-speaking world. In European and Brazilian Portuguese for instance, the distribution of rhotics is determined by syllable structure (Bonet and Mascaró 1997). A strong-R (which includes trills [r, ʀ] and rhotic fricatives [x, ɣ, χ, ʁ, h, ɦ]) is required at the beginning of words (e. g. *rua* [ʁuɐ] ‘street’) and at the beginning of syllables if the preceding syllable ends with a coda consonant (e. g. *honrado* [õˈʁadu] ‘honoured’), while a weak-r (pronounced as a flap [ɾ] in Santomean Portuguese) is required when the rhotic is the second element in an onset consonant cluster (*prato* [pɾatu] ‘plate’).2This distribution of rhotics is true of all standard varieties of Portuguese, but also of most non-standard varieties of European and Brazilian Portuguese. In coda position, the realization of the rhotic depends on various social factors, such as geographical origin and socioeconomic class. Intervocalically, the strong-R and the weak-r are phonemically contrastive (e. g. *carro* [kaʁu] ‘car’ and *caro* [kaɾu] ‘expensive’). Unlike in Brazilian and European Portuguese, a weak-r can be used in a strong-R position in Santomean Portuguese, and a strong-R can be used in a weak-r position, as shown in Table 1.

**Table 1:** Usage of the strong-R in Santomean Portuguese according to its position in a word (*n *=* *5,287; strong-R* *=* *29.7%). The different contexts refer to the positions where a strong-R, a weak-r, and neutralization are expected in standard (and most non-standard) varieties of Brazilian and European Portuguese.

Context	Position of rhotic in a word	% strong-R	*n*
Strong-R	Word-initial	69	344
	Word-medial, syllable-initial (-R)	69	313
Weak-r	Consonant cluster	15	1,746
	Word-medial, syllable-initial (-r)	34	1,597
Neutralization	Coda position	25	1,287
			Total* *=* *5,287

Bouchard (2019a: 30).

In Table 1, we see that Santomeans use a strong-R in positions that require a strong-R in Brazilian and European Portuguese only 69% of the time. This means that 31% of the time, they use a weak-r – and this is a practice that diverges from the European and Brazilian use of rhotics. In these varieties of Portuguese, the number of weak-r tokens in a strong-R position would essentially be zero. The same can be said about the use of a strong-R in weak-r positions; although the strong-R is not the most frequently used rhotic in consonant clusters (15%) and intervocalically (34%), it is often favoured by Santomeans. Based on the interchangeability of the r-sounds regardless of their position in the word, Bouchard (2017, 2019a), Pereira et al. (2018), and Agostinho et al. (2020) have argued that Santomean Portuguese might not have a phonological distinction between the weak-r and the strong-R.

**Figure 2:**
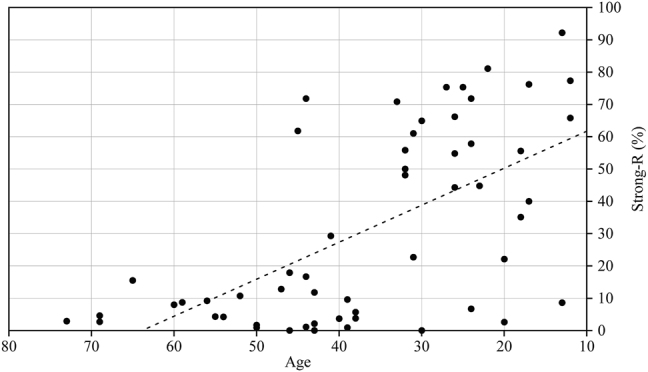
Rate of strong-R usage in Santomean Portuguese according to the speakers’ age (Adapted from Bouchard 2019a: 31).

Results in Bouchard (2019a) clearly show a change in progress in the use of rhotics, with younger generations of Santomeans using the strong-R distinctively from older generations (Figure 2). In positions that do not require a strong-R (consonant cluster, intervocalic weak-r, and coda position), the older generation never uses a strong-R. Also, in the strong-R category, the rhotic fricatives have emerged.3The most common rhotic fricative used in Santomean Portuguese is the voiced uvular fricative [ʁ] (Bouchard 2019a; Pereira et al. 2018). This is why I use the symbol [ʁ] to refer to fricative rhotics in this article. This is a phenomenon that clearly distinguishes two generations: Santomeans over 50 years of age (who do not use the rhotics fricatives) and those 49 years of age and younger (who use the rhotic fricatives). This cut-off marks the rise of the independentist movement, which was an important milestone in the history of São Tomé and Príncipe, and the formation of its national identity. Based on this observation, Bouchard (2019a) has argued that fricative rhotics also mark the emergence of an ever-developing Santomean identity.

### 2.2 The use of rhotics among the Santomean diaspora in Portugal

A high percentage of the Santomean population lives in the diaspora. The five countries that receive the greatest numbers of Santomean immigrants are Portugal, Angola, Gabon, Cape Verde, and the United Kingdom, for a total of approximately 65,000 Santomeans abroad. This might not seem like a lot, but São Tomé and Príncipe is a small country and this number corresponds to one-third of the total population of 197,700 (Instituto Nacional de Estatística 2020). With such a high percentage of Santomeans living in the diaspora, Bouchard and Desmeules-Trudel (2022) decided to investigate the use of rhotics among Santomeans living in Portugal, as it is the country with the highest number of Santomean immigrants (approximately 25,000, according to *Fórum da Diaspora Santomense* ‘Forum of the Santomean Diaspora’). We asked the following question: does migration to Portugal and closer contact with European Portuguese have an impact on the use of rhotics among Santomeans? If the marked use of rhotics (i. e., the use of a strong-R in a weak-r position) is available for identity-driven use to express a connection to São Tomé and Príncipe, then this feature should be maintained in Portugal to show identity and origin.

The results in Bouchard and Desmeules-Trudel (2022) suggest that Santomeans in Portugal are not converging towards European Portuguese in their use of rhotics. Indeed, based on an apparent-time construct, we showed that young Santomeans living in Portugal also favor the use of a strong-R over the weak-r in all word positions, and that the use of rhotics points towards an ongoing language change in Santomean Portuguese. In Table 2, we see that Santomeans in Portugal maintain their distinctive use of rhotics: the strong-R is not always used in positions that nominally require a strong-R (67% word-initially and 60% intervocalically), and it is used in positions that require a weak-r (26% consonant cluster and 43% intervocalically). Note that in most (if not all) varieties of Brazilian and European Portuguese, the use of the strong-R would be of 100% in positions that require a strong-R, and of 0% in positions that require a weak-r.

**Table 2:** Usage of the strong-R in the speech of Santomeans in Portugal, according to position in a word (*n *=* *4,048; strong-R* *=* *1,635).

Context	Position of rhotic in a word	% strong-R	*n*
Strong-R	Word-initial	67	452
	Word-medial, syllable-initial (-R)	60	327
Weak-r	Consonant cluster	26	1,165
	Word-medial, syllable-initial (-r)	43	1,181
Neutralization	Coda position	31	923
			Total = 4,048

Bouchard and Desmeules-Trudel (2022).

The authors suggest that, for some members of the Santomean diaspora in Portugal, showing affiliation with other Santomeans is more valuable or important than adapting their speech to the European practice, and that in the long term, the distinctive use of rhotics may become a symbol of this minority group in Portugal. This is consistent with Clara’s change of rhotic use, as over the course of her few years living in Portugal, her use of strong-R increased. The results of these quantitative studies on the use of rhotics provide a valuable foundation for this article; however, they do not address the processes that lead to the use and awareness of the rhotic feature, nor how this feature is being enregistered as a marker of Santomean identity.

## 3 Analytical framework and methodology

### 3.1 Enregisterment and indexicality

Existing work on enregisterment and indexicality is important to this analysis. First, this research draws on previous studies that address the enregisterment of linguistic features. In his study on the social life of cultural value, Agha (2003: 231–232) defines enregisterment as the “processes through which a linguistic repertoire becomes differentiable within a language as a socially recognized register of forms.” He focuses his work on Standard British English and how it came to be enregistered as part of a hierarchized system of speech levels and speaker rank. He highlights that the cultural values associated with linguistic features are not static, but rather the result of discursive practices, which attach cultural values to specific linguistic forms and bring these values into circulation. Enregisterment takes shape through ideological processes based on language and social values, and these values are transmitted across space and time. Following the work Agha, the concept of enregisterment has been widely adopted by sociolinguists in exploring how linguistic variation is linked with places (Johnstone et al. 2006; Johnstone and Kiesling 2008; Remlinger 2009), styles and identities (Bennett 2012; Johnstone 2017; Podesva 2011), social groups (Eberhardt 2012; Henry 2010), and standardization processes (Agha 2003; Managan 2011; Romero 2012), among others. The studies on enregisterment that focus on English-speaking communities have been particularly fruitful. Linguists have also explored other language contexts (including Indonesia [Goebel 2007]; Catalonia [Frekko 2009]; Guadeloupe [Managan 2011]; Papua New Guinea [Slotta 2012]; and Flanders [Jaspers and Van Hoof 2013], among others). However, fewer studies have been conducted in African diaspora contexts, and this paper aims to fill this gap.

Enregisterment is grounded in Silverstein’s (2003) orders of indexicality, which refer to various levels of abstraction where the relationship between linguistic expression and social meaning stabilizes. Each order of indexicality corresponds to specific criteria. Johnstone et al. (2006) combine the process linked to Silverstein’s orders of indexicality with the idea of enregisterment. In their work, they describe three orders of indexicality in terms of increasing awareness of language features, and they establish how linguistic features can become enregistered. In first-order indexicality, features usually have no social meaning for local speakers. The features are unnoticed because everyone in the speaker’s social network use them, “everybody speaks that way” (Johnstone et al. 2006: 82). But a linguist or an outsider may observe that the features correlate with a particular group – because they sound different. Features of second-order indexicality are available for social work. This means that they become ideologically linked with notions of classism, correctness, and localness (among other possible values). Speakers have become aware of the features, they attribute meaning to them, and they can use them in their own speech. One way to do social work with these features is by shifting their own styles depending on what characteristic or persona they want to express. Finally, features of the third-order indexicality have come to index a specific origin or locality, as they are increasingly perceived as markers of identity and become available to express localness. In this situation, the second-order features that index more specific notions become associated with the group at large, perpetuating the idea that places and dialects are necessarily linked. This work is made possible through speakers’ metadiscourse (explicit talk about talk). Johnstone et al. (2006) have shown that both second- and third-order features can be enregistered, and some features may be enregistered in multiple ways simultaneously, meaning that one feature can be indexical of specific qualities (second-order indexicality), but also of a place (third-order indexicality).

Using Johnstone et al. (2006) framework of three orders of indexicality, I discuss how processes of enregisterment operate on the Santomean variety of Portuguese. I narrow the focus to only one feature that is part of a shared repertoire among young Santomeans, the use of rhotics, and I suggest that it is becoming enregistered as a Santomean feature.

### 3.2 Fieldwork, data, and participants

To explore how Santomean Portuguese has become enregistered and has come to index a specific national identity, I draw on ethnographic fieldwork and sociolinguistic interviews conducted on São Tomé Island and in Portugal as part of a larger project on language use. The ethnographic fieldwork was conducted in the city of São Tomé and its surrounding areas between June 2015 and March 2017 (for a total of fifteen months), as well as in three cities of Portugal (the capital, Lisbon, and two towns in Central Portugal [*Região do Centro*]) in January 2019. Participants for this study were recruited using the friend-of-a-friend technique (Milroy 1987), which worked particularly well because the country is small and most Santomeans know many people through their large and extended social networks. In total, I recorded 118 participants on São Tomé Island and 40 in Portugal, but this study draws mainly on interviews with eight Santomeans aged between 18 and 45. Two were interviewed in São Tomé, five in Portugal, and one was interviewed in São Tomé as well as in Portugal. These eight participants were selected because their stories are representative of many other Santomeans I interviewed. The interviews were semi-structured and lasted for about 1 h. All participants were born and raised on São Tomé Island. They all speak Portuguese as their first language, and some of them also speak creole (Forro, Angolar, or Cabo Verdean) fluently. Table 3 provides a biographical and linguistic sketch of each of the participants included in this study, as well as their use of the uvular fricatives /ʁ/. This is a feature that is emerging in the speech of younger Santomeans and that is being associated with Santomean identity (Bouchard 2019a). The rate of uvular fricative use is given in Table 3 to point out the variability of this feature and to show the correlation between this feature and the participant’s year of birth (with younger Santomeans using it the most). In this paper, all eight participants are below 50 years old and are part of the population that uses the uvular fricative (Bouchard 2019a). The data were extracted from the middle of the interviews and from all phonological contexts.

**Table 3:** Participants, their story, and their use of rhotics.

Pseudonym	Year of birth	Biographical and linguistic sketch	Use of /ʁ/
Damião	1998	Damião grew up in São Tomé City, and he moved to Portugal in 2017 to study technical design. He also works part-time as a grocery store clerk to support his family and newborn child. He is monolingual in Portuguese. Damião was interviewed in Portugal in 2019.	82%
Caetano	1998	Caetano spent the first eight years of his life in the district of Caué (about 40 km from the capital), and then moved to São Tomé City with his family and attended school in the capital. He moved to Portugal in 2017 to study civil construction. Caetano speaks Portuguese, Cabo Verdean creole, and has some knowledge of Angolar and Forro. He was interviewed in Portugal in 2019.	23%
Clara	1997	Clara was interviewed twice: in São Tomé in 2015 and in Portugal in 2019. She immigrated to Portugal in 2017 to complete a professional diploma in marketing. She grew up in São Tomé City, and she is from a middle-class family. She has some knowledge of Forro.	27% (2015)
			60% (2019)
Célia	1988	Célia did a Bachelor’s degree in Public Relations in São Tomé City. She works as a journalist and a news anchor. She is a middle-class Santomean, and she is monolingual in Portuguese. She has never travelled outside of São Tomé and Príncipe. Célia was interviewed in São Tomé in 2016.	61%
Alberto	1984	Alberto was born in Ribeira Afonso (district of Cantagalo) and moved to São Tomé City when he was two years old. Alberto moved to Brazil in his twenties to study Material Science, and he lived there for seven years. He learned Forro when he was living abroad. Alberto now works for a telecommunication company in São Tomé City. He was interviewed in São Tomé in 2016.	35%
Abel	1983	Abel is from São Marçal, a suburb of São Tomé City. He is 36 years old and he moved to Portugal for his graduate studies in Law. He had been living in Portugal for two years; prior to that, he lived in Angola for two years. His partner is Angolan. Abel is bilingual Portuguese-Forro. He was interviewed in Lisbon in 2019.	87%
Aurélia	1971	Aurélia is a university student (Bachelor’s degree in Portuguese) and a primary school teacher. She is bilingual Portuguese-Forro. She lives in Oque del Rei (a suburb of São Tomé City) and has never travelled outside São Tomé Island. Aurélia was interviewed in São Tomé in 2015.	0%
Marcelo	1970	Marcelo is a businessman (forestry industry). He has spent many years in Portugal and he still visits regularly. He lives in São Tomé City and is an upper middle-class Santomean. He is bilingual Portuguese-Forro. Marcelo was interviewed in São Tomé in 2015.	38%

A thematic analysis was conducted on the participants’ narratives. During the interviews, we addressed specific topics such as language, ethnicity, social life, and their migratory experience (if any), but I attempted to let the participants lead the conversation in the directions of their choice. The parts of the interviews related to identity processes, language ideologies, language use, and belonging were transcribed. The extracts I discuss in this article are from the interviews with these eight participants.

## 4 The enregisterment of a rhotic feature in Santomean Portuguese

### 4.1 First-order situation and the variability of rhotics in Santomean Portuguese

São Tomé Island is isolated, and most Santomeans have no face-to-face contact with people who speak Portuguese differently than they do. They are part of dense, multiplex social networks (in Milroy’s 1987 terms) that strengthened the local variety of Portuguese. In their interviews with me, many Santomeans (especially older Santomeans) mentioned or insinuated that European Portuguese is perceived as the standard and most prestigious variety. This is an ideology that has been circulating among Santomeans most likely since the second phase of colonization (1850–1975) when the transmitted belief was that Portuguese was “better” than the local creole languages (Bouchard 2019b). This is common in the history of colonial and creole societies; for instance, English is still, in many ways, the language of prestige in Jamaica (Wassink 1999), as is French in Haiti (Hebblethwaite 2012), and Dutch in Aruba, Bonaire, and Curaçao (Dijkhoff and Pereira 2010). Nowadays, contact with European Portuguese in São Tomé and Príncipe mainly comes from broadcasting and the internet. A small number of Portuguese nationals live and work on the island, mainly in the embassy, NGO’s, the United Nations, private and family businesses, schools, cultural centers, and churches. Most Portuguese and other non-Portuguese whites (mainly from Brazil and France) live in the capital, in privileged houses and neighbourhoods. Contact with European Portuguese, even if infrequent or indirect, means that a number of linguistic features that are characteristic of Santomean Portuguese can be heard as nonstandard. Among these linguistic features are the monophthongization of diphthongs (Christofoletti 2013), the absence of nominal and verbal agreement (Brandão 2011a, 2011b; Brandão and Vieira 2012; Figueiredo 2008), different strategies for wh-constructions and clefts (Gonçalves 2013), and the absence of articles (D’Apresentação 2013; Espírito Santo 1998; Lorenzino 1996). The first-order indexicality of these forms has been brought into second-order indexicality, as the use of most of these local linguistic features correlates with living setting, since Santomeans living in rural areas use these forms more often. But the case of the rhotics is slightly different as it does not correlate with rural settings; most speakers who show a higher rate of strong-R in their speech come from the capital and are young.

It is in the societal transition from creole to Portuguese that the fricative rhotics have emerged in Santomean Portuguese. The fricative rhotics, produced in the back of the vocal tract, are replacing the alveolar trill, pronounced in the front of the vocal tract – a changed that has already happened in Portugal (Veloso 2015). Bouchard (2019a) has demonstrated that Santomeans show great variability in their use of rhotics. However, most Santomeans living on the islands are not aware that their use of rhotics is different from the rest of the Portuguese-speaking world. During the interviews I conducted on São Tomé Island, it was difficult (and sometimes impossible) to collect metalinguistic information about the use of rhotics in the participants’ speech or in the community at large. Many participants did not even know what I was talking about. The few participants who mentioned the r-sounds as a local feature were people from a higher socioeconomic status who had studied or worked abroad, and people who had come into contact with Portuguese or Brazilians. While some Santomeans had greater metalinguistic awareness, most could not explain what exactly the differences were nor describe their use of rhotics, but they knew they pronounced the r-sounds differently from other Portuguese speakers because someone had told them so. An example of this comes from an interview with Aurélia, born in 1971 (44 years old at time of interview), who works as a school-teacher and studies Portuguese linguistics at the undergraduate level at a university in São Tomé City and who did not pronounce any strong-R during her interview (Table 3). Aurélia talked a lot about language use in her interview, but the use of rhotics did not come up. The focus was mainly on Portuguese and Forro and how these two languages are used in her surroundings. In the fiftieth minute of the interview, I asked Aurélia if she noticed anything special in the use of rhotics in the speech of young Santomeans. Because she is a highly educated Santomean who works in education, I was hoping to access information that differed from my own observations. But I did not succeed, as the following excerpt shows:

Marie-Eve: *Você reparou o erre dos mais jovens?*

Aurélia: *Não*.

Marie-Eve: *Os mais jovem falam … em vez de falar “brasileiro” [brɐzilɐjru], eles falam “brasileiro” [bʁɐzilɐjʁu]. Reparou?*

Aurélia: *Não, mas sempre a professora Renata também sempre dizia isso, que nosso erre é muito diferente, tá a ver.*

Marie-Eve: *Eu reparei porque pra mim é muito diferente, mas vocês aqui …*

Aurélia: *Ninguém repara! (Risos) Nós não damos por isso! (Risos)*

Marie-Eve: *Quase todas as crianças falam assim, com esse erre. Agora estava no mercado e ouvi uma senhora gritar “farinha, fariiiinha” (pronounced [ fɐʁiɲɐ]).*

Aurélia: *(Risos)*

Marie-Eve: *Em vez de falar “farinha” (pronounced [fɐɾiɲɐ]).*

Aurélia: *Farinha (pronounced [fɐʁiɲɐ]).*

Marie-Eve: Have you noticed how the youths pronounce the R?

Aurélia: No.

Marie-Eve: The youngest say … instead of saying Brazilian [brɐzilɐjru], they say [bʁɐzilɐjʁu]. Did you notice?

Aurélia: No, but Professor Renata would always say this as well, that our R is very different, you see.

Marie-Eve: I noticed because for me, this is very different, but you here …

Aurélia: Nobody notices that! (Laughs) We don’t care about that! (Laughs)

Marie-Eve: Almost all children speak like this, with this R. Right now, I was at the market and a lady was shouting *farinha, fariiiinha* ‘flour, floouuur’ (pronounced [fɐʁiɲɐ]).

Aurélia: (Laughs)

Marie-Eve: Instead of *farinha* ‘flour’ (pronounced [fɐɾiɲɐ]).

Aurélia: *Farinha* ‘flour’ (pronounced [fɐʁiɲɐ]).

Aurélia is aware that Santomeans use the r-sounds differently because one of her university professors was Brazilian and they discussed this distinction in class (*a professora Renata sempre dizia que nosso R é muito diferente* ‘Professor Renata would always say that our Rs are very different’). Aurélia could repeat comments and explanations that came from her professor, but she did not discuss this matter any further with me. For Aurélia, the distinctive use of rhotics has not been enregistered. Even if the social facts show that this particular use of the rhotics indexes the youth and the post-independence period – it was obvious to my ears and to professor Renata – this connection was not observable in Aurélia’s surroundings. And this was the case for most Santomeans over age 50.

This distinctive use of the rhotics is quite recent, and it only shows up in the speech of Santomeans born after the country’s independence (1975) (Bouchard 2019a; Pereira et al. 2018). It is a feature with potential first-order indexicality that has not yet come to the level of consciousness of many Santomeans. But as it is heard more and more in the speech of young Santomeans, it acquires social meaning and results in second-order sociolinguistic work, connecting with incorrectness, youthfulness, and locality.

### 4.2 Second-order indexicality and the perception of the rhotic feature as problematic

In a second-order situation, speakers begin to notice the features they use and ascribe to them some kind of local meaning. For Santomeans, contact with non-Santomeans is key to bringing their distinctive use of rhotics to their awareness. As I mentioned earlier, the only Santomeans I have met on São Tomé Island who were able to metadiscoursively examine their use of rhotics were people who had been in close contact with speakers of other varieties of Portuguese. During the interviews, the participants introduced labels for their ways of pronouncing rhotics that differed from the norm. Most commonly, their use of rhotics is referred to as *carregar nos erres*. *Carregar* in this sense means ‘to turn stronger or more intense’, and *erre* stands for the letter R. Many Santomeans refer to this distinctive pronunciation of rhotics as a “problem” and a “defect of language.” This represents a common opinion I have heard regarding the distribution of rhotic variants in the Portuguese spoken by Santomeans. After receiving comments on their use of rhotics, some Santomeans that I interviewed had tried different techniques to “remove” this pronunciation, such as Alberto, a 32-year-old Santomean who studied in Brazil:

*Eu percebo que eu carrego no erre. […] Já tentei, já fiz tipo exercícios com amigos conhecidos de dicção para tentar retirar o erre, mas depois parei (risos) e acabei desistindo.*

I notice that I make my R stronger. […] I tried, I did some diction exercises with friends to try to remove this R, but then I stopped (laughs) and I gave up.

Alberto has come to accept the way he speaks, although he knows there is something different about his pronunciation. This Santomean use of rhotics is often linked in discourse with incorrectness, usually in comparison with European Portuguese. Even if Santomeans are sometimes mocked for their use of rhotics, this feature remains available and is used for social work. As mentioned in Bouchard (2018), it is important to point out how this idea that the Santomean way of pronouncing rhotics is incorrect or different comes from contact with speakers of other varieties of Portuguese. During my stay in São Tomé, I never heard any Santomeans discussing, mocking, or criticizing another Santomean’s pronunciation of rhotics.

Célia is a 27-year-old Santomean who also perceives her use of rhotics as incorrect. She is a strong-R user and she has become aware of this feature through her work as a news anchor at STP News.4STP News is a pseudonym for the journal where Célia works. Célia grew up in Riboque, a lower- to middle-class neighbourhood that is centrally located in São Tomé City. She has spent her entire life living in the capital, where she attended primary school, high school, and university. She has never travelled outside São Tomé Island. The news videos that she produces are available online, and people can freely comment on her speech. The following is an example of comments that someone posted on the STP News Facebook page a few days before I interviewed her:

*Essa jornalista por amor a santa, não tem a categoria que faltou na reportagem, uma boa oratória, pelo contrário, padece de um grande desvio fonético, precisa urgente de uma boa terapia intensiva de fala.*

This journalist, for heaven’s sake, she does not have what is needed for this news report, good public speaking skills, on the contrary, she suffers from a grave phonetic deviation, she urgently needs good intensive speech therapy.

(Comment discussing Célia’s speech on Facebook, February 12, 2016.)

Before reading the comments that people write on the STP News Facebook page about her speech, Célia was unaware of the values (of (in)correctness in this case) that others might associate with the way she speaks, mainly because she was not in contact with people for whom her pronunciation of rhotics could carry second-order indexical meaning. But through repeated occurrences of pejorative comments based on her accent, Célia learned to hear her accent as socially stigmatized. According to her, her social environment explains her pronunciation of rhotics:

*Eu vim de um círculo muito pobre e não tive nenhuma influência de pessoa que fala português de Portugal [é] então a minha mãe fala assim, as minhas irmãs falam assim, as pessoas ao pé de mim, o meu círculo familiar fala assim, o meu companheiro ele não fala assim porque também viveu muito tempo em Cuba [ok] então a pronúncia mudou e tal, então eu acho que os Santomenses que são Santomenses, particularmente de um círculo baixo né, um nível social baixo que eh … que nunca saíram daqui nunca tiveram outro tipo de convivência e tal, convivência direita [sim] falam assim como eu.*

I come from a very poor social circle and I didn’t have any contact with people who speak Portuguese from Portugal [yeah] so my mom speaks like this, my sisters speak like this, people close to me, my family speaks like this, my partner does not though because he lived in Cuba for a long time [ok] so his pronunciation changed and everything, so I think that Santomeans who are Santomeans, especially the ones from a low social circle, a lower social class, that … who never travelled outside São Tomé, who never had any other kind of interaction and all, direct interaction [yes] they talk like me.

Célia links her distinctive pronunciation of rhotics to Santomean identity (*os Santomenses que são Santomenses* ‘Santomeans who are Santomeans’), more specifically to Santomeans from a lower socioeconomic status who have not interacted with speakers of Portuguese who are not Santomean. However, this information should be provided with some context; although Célia might come from what she considers to be lower socioeconomic class, she now has a better life. In my opinion, she is part of a middle socioeconomic class (according to Santomean standards). She has a high level of education, good employment, and owns a house with her partner. Also, her mother (with whom I interacted a few times) is not a strong-R user, even if Célia claimed *a minha mãe fala assim* “my mom speaks like this.” She might have been referring to other linguistic features that characterise the Santomean accent. That being said, in this interaction above, social meaning is attributed to the use of rhotics. The strong-R still correlates with age (first-order indexicality), but the feature is also available to point to localness and social status (second-order indexicality). The excerpt above shows that even if the use of strong-R is perceived as incorrect by outsiders, it is linked by Célia (as by other Santomeans when they become aware of it) to a shared localness.

As mentioned earlier, one way to do social work with rhotics is by using different r-sounds depending on what characteristic or persona the speaker wants to express. Abel, who lives in Portugal, gives an example of this in the following excerpt, in which he explains that his use of rhotics varies according to his interlocutor:

*Marie-Eve: O que eu acho interessante é que não são todos os Santomenses que carregam no erre.*

*Abel: Sim, sim, não são todos, verdade é, não são todos. Eu próprio tenho essa dificuldade às vezes de R, às vezes carrego às vezes não. Quando estou a falar perante alguém que eu preciso manter um nível cuidado, eu controlo, mas quando não, é so falar é so falar.*

Marie-Eve: What I find interesting is that not all Santomeans turn their R strong.

Abel: Yes, yes, not all of them, that’s true, not all of them. I myself have this problem with the Rs sometimes, sometimes I turn them strong and sometimes I don’t. If I’m talking with someone with whom I have to maintain a certain level of carefulness, I control it, but when not, I just talk and talk.

Abel expresses awareness that one form is perceived as more “correct” than the other one, and that different social meanings are attached to the different uses of rhotics. In this case, Abel claims that he can “control” his use of the rhotics and that he is more careful in a formal context or when interacting with someone that he perceives as being of high social status. In an informal context or with people with whom he does not consider that he has to be careful about his speech, he does not pay attention to his use of the r-sounds. What is interesting here is not so much Abel’s ability to alternate between different uses of rhotics according to his interlocutor and the context, but rather his explanation regarding style shifting and how he connects “control” with a more standard use of the rhotics, as well as with a more formal context or interlocutor. Interestingly, this awareness of the different uses of the rhotics and their social meanings does not mean that Abel (as other Santomeans) can actually “control” their use of rhotics. Even when Abel was explaining to me the difference between the pronunciation of a strong-R and a weak-r in the interview, he pronounced the two forms of rhotics that he was contrasting with an uvular fricative (intervocalically in *correr* ‘to run’ versus word-medially in coda position in *arca* ‘chest’). Note that the word-final rhotic in *correr* was also pronounced with a uvular fricative.

*Não consegue fazer essa distinção entre ‘correr’ [kuʁeʁ] e por exemple ‘arca’ [aʁkɐ], tá a ver, um carrega-se mais outro fica-se mais … mais leve não é, e a gente muitas vezes não faz essa distinção, a gente quando é erre, é pronunciar só.*

They can’t make the distinction between *correr* [kuʁeʁ] ‘to run’ and for example *arca* [aʁkɐ] ‘chest’, you see, one is stronger and the other one is more … is lighter, right, and often we don’t make this distinction, when there’s an R we just say it.

Based on my observations and on previous work on Santomean rhotics (Bouchard 2019a; Brandão 2016; Pereira et al. 2018), Santomean Portuguese lacks a phonological distinction between the strong-R and the weak-r. This could explain the difficulty to perform the phonemic distinction between the strong-R and the weak-r. Even so, in discourse, the r-sounds in Santomean Portuguese are available to mark localness, social status, and correctness (second-order indexicality).

### 4.3 Migration to Portugal and third-order indexicality

Third-order indexicality entails explicit metadiscourse about the use of rhotics, and it points to associations with other ideological meanings. In other words, in a third-order situation, the features associated with special social values or social categories are the object of comments, the level of awareness is at the highest, and speakers can deliberately use the features to perform identity. Based on fieldwork among Santomeans on São Tomé Island and in Portugal, I believe that part of the third-order indexicality happens abroad. In the mid-1970s, the immigration flow towards Portugal intensified with the independence of the former Portuguese colonies in Africa, including São Tomé and Príncipe in 1975. And as mentioned earlier, Portugal is the country that receives the greatest number of Santomean immigrants. Most of the 25,000 Santomeans living in Portugal remain in contact with their families and friends on the island. With such a high number of Santomeans in Portugal, it is important to include them in the studies on Santomean Portuguese as they might have a role to play in the variation and change of different linguistic features.

The second-order indexicality of the rhotic feature and its potential to indicate age, informality, and local identity becomes available via those Santomeans who are in contact with other Portuguese speakers, those who immigrated abroad, or those who lived abroad for a certain period of time and came back to São Tomé. In this context, the distinctive use of rhotics comes to index Santomean national identity, and not necessarily youthfulness or social status. In their discourse about language use, Santomeans who live in Portugal can easily express the distinction between the Santomean and European varieties of Portuguese, in comparison with Santomeans who live on the island. This is mainly due to the comments they receive about their accent. For many, European Portuguese is still perceived as more prestigious, as it is the main variety spoken in their host country, and their use of rhotics is still considered to be problematic, as Caetano mentioned in the following excerpt:

Marie-Eve: *Quais são as diferenças entre o português de cá e o português de* *São Tomé?*

Caetano: *Português de São Tomé, acho que português de São Tomé nós … tipo, nós temos um problema que é carregar os erres [hm hmm] nós falamos mais a carregar os erres mas o sotaque de Portugal, fala mesmo normal*.

Marie-Eve: What are the differences between Portuguese here and Portuguese in São Tomé?

Caetano: Portuguese from São Tomé, I think that the Portuguese from São Tomé we … like, we have that problem that we draw out the Rs [hm hmm], we turn the R strong when we talk, but the accent from Portugal, they speak normal.

We see in this excerpt that in explicit metapragmatic discourse, the pronunciation of rhotics in European Portuguese is perceived as normal, while in Santomean Portuguese, it is still perceived as problematic. But regardless of such pejorative discourse among Santomeans and regardless of the comments that Portuguese nationals might give them, I was surprised to observe the high rate of strong-R usage in the speech of the young Santomeans in Portugal. The case of Clara (Figure 1) is a prime example of this. I believe that this use of the strong-R is related to a growing awareness of this distinctive feature, as well as a sense of pride that comes with contact in diasporic communities. In fact, many Santomean participants have expressed pride in their national identity and in their ways of speaking. This is illustrated in the interview excerpt below, with Damião, a 21-year-old Santomean living in Portugal.

*Mesmo que chega no momento que alguém me dizer, “ah você fala muito mal o português”, não, isso é uma coisa que eu … nasci com ele vou morrer com ele, é … é uma das coisas que consigo ter, consegui trazer até aqui de São Tomé, vou morrer com isso, meu sotaque, minha maneira de falar.*

Even if someone at some point tells me “Ah, you speak bad Portuguese”, no, that’s a thing that … I was born with it and I’ll die with it, it’s … it’s one of the things that I was able to have, able to bring here from São Tomé, I’ll die with it, my accent, my way of speaking.

As is the case for many other Santomeans I interviewed, contact with Portuguese nationals and other Portuguese-speaking Africans in Portugal have made them aware of their accent and their distinctive use of rhotics, and it awoke a feeling of national identity. Others, however, do change their accent and converge toward the European forms. In the following excerpt from Célia, we see how a Portuguese teacher recognizes the Santomean accent and considers it to be *estranho* ‘weird’, and also how some Santomeans in Portugal perceive negatively the use of rhotics in Santomean Portuguese.

*O meu colega, nós estavamos a falar da pronúncia ainda anteontem, ele foi pra Portugal no final do ano passado, foi estudar, fazer licenciatura, e ele diz que a professora dele de português disse a ele que nos Santomenses falamos duma forma muito estranha, só que o tom que ela usou para falar isso a ele, ele não gostou [é] ele sentiu que ela estava a menosprezar a forma como nós falamos [é] mas a professora não deixou ele tão irritado assim quanto um amigo santomense que já está em Portugal há algum tempo e fala como os Portugueses falam, ele estranhou a forma como meu amigo fala de uma forma que deixou meu amigo bem chateado porque alem dele falar a ele que ele fala mal fala diferente, ele falou que carrega muito no erre de uma forma estranha [ok] e o meu amigo aborreceu mais com o colega dele que é Santomense porque ele é Santomense, ele falava assim antes de sair daqui, e so porque agora tá la fala como os Portuguese falam ele (risos).*

With my colleague, we were talking about pronunciation the day before yesterday, he went to Portugal at the end of last year, he went to study, to do a Bachelor’s degree, and he said that his Portuguese teacher told him that us Santomeans we speak in a weird way, but the tone that she used when she told him that, he didn’t like that [ok] he felt like she was belittling the way we speak [ok] but his teacher didn’t make him as mad as his Santomean friend who has been in Portugal for a while and speaks like the Portuguese do, he was surprised to hear the way my friend speaks and that made my friend angry because on top of telling him that he speaks bad, that he speaks differently, he said that my friend turns his R strong in a weird way [ok] and my friend got more mad at him because he’s Santomean, he used to speak like this before leaving the island, and now because he’s there he speaks like the Portuguese (laughs).

For Portuguese nationals who have been in contact with Santomeans, this distinctive use of the rhotics is noticeable and obvious. They recognize Santomeans by their use of the rhotics, and as indicated in this excerpt, their accent can be perceived as “weird”. Once there is a cultural story that ties together a linguistic feature with a group (or a place), this feature can become enregistered as pertaining to that group. A strong-R in a phonemic weak-r position is such a distinctive feature of Santomean Portuguese that non-Santomeans (in São Tomé and in Portugal) use the strong-R in all environments when mocking Santomeans – something that I have heard many times. There is some kind of overgeneralization in the perception of the rhotics that can lead to generalization, as is the case in a footnote of Veloso’s (2015: 333) article on the Portuguese rhotics: “This is the case of some varieties spoken around the Portuguese city of Setúbal (Southern dialects) and of São Tomé Portuguese (STP), where flaps do not exist at all” – meaning that the strong-R is used all the time, which is not the case. In Labov’s (1972) terms, this Santomean use of rhotics is a stereotype. Interestingly, in the excerpt above, the Santomean friend was more annoyed with his compatriot for commenting on his accent than he was with his Portuguese teacher. This is not surprising – as the rhotic feature is becoming a marker of Santomean identity, using this feature can show belonging to the group, and not using it or criticizing it can be perceived as an act of non-belonging. Through the process of enregisterment, repeated use of the rhotic feature allows Santomeans to identify themselves with this feature, and then use it to reinforce a group identity. In the excerpt above, Célia interprets the friend’s negative evaluation of the rhotic feature as a desire to sound more Portuguese – and at the same time, as an act of non-belonging.

The following table (Table 4) summarizes the enregisterment process of the rhotics in Santomean Portuguese.

**Table 4:** Orders of indexicality, description, and application to Santomean Portuguese.

Orders of indexicality	Description (cf. Silverstein 2003; Johnstone et al. 2006)	Application to the use of rhotics in Santomean Portuguese
First-order indexicality	Features might not have any social meaning for local speakers; they are unnoticed because everyone in the speaker’s social network use them. But an outsider may observe that the features correlate with a particular group.	Great variability in the use of rhotics, especially among young Santomeans from the capital and its surrounding areas, but most Santomeans living on the island are unaware of it.

Second-order indexicality	Speakers have become aware of these variable features, they attribute meaning to them, and they can use them in their own speech to do social work (by style shifting, for instance).	Santomeans become consciously aware of this feature when they are in contact with non-Santomeans (on the island or in the diaspora).
		Some Santomeans consider this feature (the *R puxado* ‘strong-R’) to be problematic and incorrect, while others think it marks localness.

Third-order indexicality	Features have come to index localness, as they are increasingly perceived as markers of local identity. This is made possible through speakers’ metadiscourse.	In Portugal, the use of the strong-R is becoming recognized as a marker of Santomean identity.

## 5 Discussion and conclusion

I now return to Clara and her use of rhotics, as it is the change in Clara’s speech from 2015 to 2019 (Figure 1) as well as all the Santomeans who have asked me “where does this R come from?” that inspired this work on indexicality and enregisterment. How does Clara fit into this bigger picture? In 2015, Clara seemed unaware of her distinctive use of rhotics even if she already had a high percentage of uvular fricative usage in her speech. Four years later, Clara had become aware of this feature, as shown in the starting quotation at the beginning of this article, where she refers to Portuguese nationals who say that Santomeans sound like French speakers because of their pronunciation of rhotics. The fact that she was living in Portugal and was in everyday contact with European Portuguese (as well as other varieties of African Portuguese spoken by friends and colleagues in her social network) did not lead to a more standard use of rhotics. This leads me to suggest that certain social values get attached to rhotics in Santomean Portuguese and European Portuguese on São Tomé Island, and then these get enregistered in a particular way in the diaspora, where the rhotic feature is used (consciously or not) to mark Santomean national identity. The observation that language contact in the diasporic community pushes the enregisterment process is one important contribution of this study for theories of enregisterment, as most existing studies focus on language use in the home communities. Another contribution is that, rather than analysing the rhotic feature as an object of sociolinguistic observation – which has been the case so far in the literature on rhotics in Santomean Portuguese – it is analysed in terms of its potential use in social work. This situates the study in the third wave of sociolinguistic research, in which variation is perceived as essential to language and a reflection of social identities (cf. Eckert 2012).

That being said, the process of enregisterment of the rhotic feature in Santomean Portuguese is not straightforward; on one hand, it takes time for the ideological process that links the rhotic feature and social values to become visible and recognizable, and on the other hand, some group members resist the change in the use of rhotics in Santomean Portuguese by adopting the European standard. Even so, the present study and previous work on rhoticity in Santomean Portuguese (Bouchard 2019a; Bouchard and Desmeules-Trudel 2022) indicate that the distinctive use of rhotics is becoming a marker of Santomean identity in the diasporic context of Portugal.

In this article, I have described three different stages in the process of sociolinguistic enregisterment in order to discuss how the ways of pronouncing rhotics came to identify Santomeans and how they are being enregistered in terms of metalinguistic commentary. I have focused on the explicit association of a distinct use of rhotics with the Santomean identity within the Portuguese-speaking world. As a result of the enregisterment process, Santomeans are able to invoke this specific linguistic feature to mark a particular identity. The use of rhotics in Santomean Portuguese is of particular interest because it differs from the other linguistic features that can be associated with this variety of Portuguese. While other features have been enregistered as markers of rural and creole identity since colonial times, the distinctive use of rhotics is a new feature that is still emerging in Santomean Portuguese. The growing use of this rhotic feature is contributing to its enregisterment, and the indexical value of the linguistic feature is being created. The development and maintenance of an enregistered feature involve the speakers’ overtly explicit evaluations of the feature (third-order indexicality) as well as its use to mark a specific social group or identity (second-order indexicality). This is currently observable in the speech of Santomeans who have been in contact with Portuguese-speakers from Portugal or Brazil. Santomeans who have not been in contact with non-Santomeans did not mention anything about the rhotic feature during the interviews and could not answer my questions related to this feature. This feature is unnoticeable to them – discussions with them about language focused on the use of creole rather than on specific features of Portuguese.

The most interesting finding in this study is that the growing awareness of this rhotic feature among Santomeans is contingent on having contact with Portuguese speakers of non-Santomean origin. Santomeans only become aware of their distinctive use of rhotics when they are in contact with speakers of another variety of Portuguese on the island or in the diaspora. Also, even if this feature is perceived negatively by non-Santomeans (and by some Santomeans as well), it remains available for identity-driven use to express a connection to São Tomé and Príncipe. This study contributes to the theories of enregisterment by including a speech community of a tiny nation-state and its diaspora to the conversation, and by demonstrating that members in the diaspora may also play a role in the enregisterment process, which continues and is refashioned when speakers move abroad.
